# Continuing medical education

**DOI:** 10.4103/0019-5545.43631

**Published:** 2008

**Authors:** Chittaranjan Andrade

**Affiliations:** Department of Psychopharmacology, National Institute of Mental Health and Neurosciences, Bangalore 560 029, India

## CME QUESTIONS

- Many meta-analyses have recently examined the efficacy of antidepressants in major depressive illness. With this background, mark *True* or *False* against each of the following statements:
  1. In adults with major depressive disorder, antidepressant drugs are more effective when depression is severe than when it is mild.
  2. In patients with major depressive disorder, antidepressant drugs are less effective in studies that are placebo-controlled.
  3. In adults with major depressive disorder, the selective serotonin reuptake inhibitors (SSRIs) are comparable in efficacy.
  4. Dual-acting antidepressant drugs are superior to the SSRIs.
- Advances in medicine and improved healthcare delivery systems have together increased the number of persons who survive into old age. Geriatric psychiatry, and particularly the treatment of the cognitive problems of the elderly, have become important areas of current research. In this context, several novel approaches have been studied towards the prevention or treatment of cognitive deficits in the elderly, in those with mild cognitive impairment, and in those with dementia. With this background, mark *True* or *False* against each of the following statements:
  1. The nonsteroidal antiinflammatory drugs (NSAIDs) naproxen and celecoxib prevent cognitive deterioration in old age.
  2. Statins may protect against incident dementia.
  3. Omega-3 fatty acids improve cognition in the elderly.
  4. Disease-modifying treatments for Alzheimer's disease are beyond reach.
  5. Diverting CSF away from the brain can reduce the exposure of the brain to amyloid and tau; this novel approach can benefit patients with Alzheimer's disease.

## CME ANSWERS

### A) Efficacy of antidepressant drugs in major depressive disorder

Answers: 1. False; 2. True; 3. False; 4. True.

#### 1. Depression severity and antidepressant efficacy

Kirsch *et al*.[1] described a meta-analysis of complete datasets on all trials (*n* = 47) of newer antidepressant drugs in major depressive disorder, submitted for regulatory approval to the Food and Drug Administration in the USA. The final analysis was based on 35 placebo-controlled trials of fluoxetine, venlafaxine, nefazodone, and paroxetine. Kirsch *et al.* found that the response to placebo was almost as good as the response to antidepressants when depression was mild; however, the placebo response decreased as the severity of depression increased. Therefore, antidepressant medication was significantly superior to placebo only when depression was more severe. This finding notwithstanding, the efficacy of antidepressant drugs was quite uniform across the spectrum of severity of depressive illness.

#### 2. Antidepressant efficacy in placebo-controlled studies

In a meta-analysis of studies in geriatric depression, Sneed *et al*.[2] showed that the antidepressant response rates were lower in trials which compared antidepressant with placebo than in trials which compared two antidepressants. Thus, just as the response to placebo is enhanced by a belief that the medication is effective, the response to antidepressant medication is diminished if patients think that they may be receiving placebo.

#### 3. Comparative efficacy of selective serotonin reuptake inhibitors

Kennedy *et al*.[3] meta-analyzed all randomized controlled trials in which escitalopram (10-20 mg/day) had been compared with other antidepressant drugs in adults with major depressive disorder. There were ten trials; these compared escitalopram with citalopram (20-40 mg/day), fluoxetine (20-40 mg/day), paroxetine (20-40 mg/day), sertraline (50-150 mg/day), and venlafaxine (75-225 mg/day). Escitalopram was found to be as effective as venlafaxine and slightly more effective than the other SSRIs. The advantage for escitalopram was more apparent in more severely depressed patients and was most evident at the 20 mg/day dose. The adverse event drop out rate did not differ between escitalopram and the other SSRIs but was lower with escitalopram than with venlafaxine. Interestingly, in a more recent, pooled analysis of two randomized controlled trials,[4] escitalopram (10-20 mg/day) was associated with higher response and remission rates, and with lower drop out rates, than the dual acting antidepressant duloxetine (60 mg/day) in patients with major depressive disorder; the superiority of escitalopram over duloxetine was evident even in a subset of patients with severe depression.

#### 4. Dual-acting drugs vs. SSRIs

Papakostas and Fava[5] found that, its dual mechanism of action notwithstanding, milnacipran (100-200 mg/day) was not more effective than fluoxetine (20 mg/day), fluvoxamine (200 mg/day), or paroxetine (20 mg/day) in adults with major depressive disorder (however, its dual mechanism of action notwithstanding, milnacipran was not associated with more dropouts than these SSRIs). In the pooled analysis referred earlier,[4] escitalopram was found to be superior to the dual-acting drug duloxetine. The controversies associated with the subject notwithstanding, most data do support the advantage for dual-acting drugs over the SSRIs. A very recent meta-analysis[6] found that the advantage is significant but small; the number needed to treat was 24, meaning that 24 depressed patients would need to be treated with a dual-acting drug (as opposed to an SSRI) for one additional patient to respond.

### B) Novel approaches to the treatment of cognitive states in the elderly

Answers: 1. False; 2. True; 3. False; 4. False; 5. False.

#### 1. Nonsteroidal antiinflammatory drugs

A large body of epidemiological literature suggests that the long-term use of NSAIDs in conditions such as arthritis is associated with a decreased risk of Alzheimer's disease.[7,8] However, in a large (*n* = 2528), randomized, double-blind, placebo-controlled trial, the Alzheimer's disease anti-inflammatory prevention trial (ADAPT) Research Group[9,10] found that two years of treatment with naproxen (220 mg twice daily) or celecoxib (200 mg twice daily) did not protect against cognitive decline in cognitively healthy men and women aged 70 years and older; these drugs, in fact, appeared to worsen cognitive outcomes.[9] Furthermore, naproxen and celecoxib either did not prevent or actually increased the risk of Alzheimer's disease.[10]

There is no logical reason to suspect that, in the epidemiological studies,[7,8] cognitively healthier patients were more likely to receive NSAID treatment for arthritis, or that arthritis protected against Alzheimer's disease. So, if NSAIDs truly protect against Alzheimer's disease, why were the results of the ADAPT study[9,10] negative? One reason could relate to the drugs and doses examined; for example, indomethacin and ibuprofen may be neuroprotective whereas naproxen and celecoxib are not. Another reason could be that the window of opportunity was lost in the ADAPT study; NSAIDs may be neuroprotective in younger subjects, before the neuropathology of Alzheimer's disease is established.

It must be remembered here that a balance of mechanisms may be involved. NSAIDs may impair cognition because, by inhibiting cyclooxygenase mechanisms, they interfere with glutamate-dependent learning and memory;[11,12] but these drugs may protect against Alzheimer's disease by inhibiting the neurodegeneration resultant from the inflammatory response to amyloid. The mechanism which predominates may depend on the drug and the state of the brain at the period of administration.

#### 2. Statins

Some epidemiological data suggest that the use of statins is associated with a decreased risk of incident dementia.[13,14] In another, very recent, epidemiological study (*n* = 1789), Cramer *et al*.[15] found that, across a 5-year follow-up period, cognitively healthy elderly subjects who used statins enjoyed a halved risk of dementia or cognitive impairment without dementia.

How may statins reduce the risk of Alzheimer's disease? The enzymes beta- and gamma-secretase cleave amyloid precursor protein and form amyloid-β, which in turn forms the amyloid plaque that characterizes Alzheimer's disease. Only a small quantity of amyloid precursor protein follows this pathway; the rest is cleaved by alpha-secretase to form non-toxic products. Statins inhibit beta-secretase and activate alpha secretase. In animal studies, statins have been shown to reduce amyloid-β levels.[16]

It should be remembered that statins may also reduce the risk of dementia and cognitive impairment by modifying the vascular risk factors that have been implicated in both vascular dementia and Alzheimer's disease.

#### 3. Omega-3 fatty acids

Epidemiological studies suggest that fish intake reduces the risk of age-related cognitive decline and Alzheimer's disease. For example, Morris *et al*.[17] found that the dietary intake of omega-3 fatty acids and an at least once-weekly consumption of fish decreased the risk of Alzheimer's disease. Huang *et al*.[18] found that elderly subjects who ate fatty fish more often than twice a week had a 41% lower risk of Alzheimer's disease; though, the benefits were limited to subjects without the apolipoprotein E ε4 allele. Nurk *et al*.[19] showed that elderly subjects who ate more fish and fish products had better cognitive performance across a range of cognitive domains; the effect was dose-dependent. Van Gelder *et al*.[20] showed that elderly subjects who ate fish and who consumed more omega-3 fatty acids in their diet suffered less cognitive decline across 5 years; the effect was again dose-dependent.

The benefits of fish are believed to arise from the omega-3 polyunsaturated fatty acid content. However, a recent, large (*n* = 302), 26-week, randomized, double-blind, placebo-controlled trial[21] found that eicosapentaenoic acid and docosahexaenoic acid supplementation in the dose of up to 1800 mg/day did not improve cognitive measures in cognitively intact elderly subjects.

How can one reconcile the contrast between the epidemiological data and the findings of this randomized controlled trial? Epidemiological studies report on behavior across years or even decades; if fish oil supplementation is at all beneficial, it may need to be sustained for long periods for cognitive benefits to be detectable. Thought must also be given to the importance of beginning healthy behavior at a younger age, when the biological capacity to respond to beneficial influences is greater, than at a later age, when systems are losing their flexibility.

#### 4. Disease-modifying treatments for Alzheimer's disease

Statins have already been discussed as potential disease-modifying treatment for Alzheimer's disease. Nearly a dozen other drugs are currently under development. For example, immunological approaches that prevent the conversion of amyloid-β into pathological forms and approaches that accelerate the clearance of amyloid-β are presently under study. The challenge is to establish a favorable balance between the risks associated with the induction of an autoimmune reaction and the benefits associated with the immunological clearance of a potentially harmful endogenous protein

PBT2 is a potentially disease-modifying drug. In Alzheimer's disease, the conversion of the amyloid-β peptide from a physiological, water-soluble, monomeric form into neurotoxic oligomeric and fibrillar forms is an undesirable occurrence.[22] PBT2 is a metal-protein attenuating compound that reduces the copper- and zinc-mediated toxic oligomerization of amyloid-β. Animal data from transgenic mouse models of Alzheimer's disease suggest that PBT2 may be beneficial in Alzheimer's patients. A Phase IIa clinical trial[23] found that, in 78 patients with early Alzheimer's disease, 12 weeks of treatment with PBT2 (250 mg/day) was associated with reduced levels of cerebrospinal fluid (CSF) amyloid-β42 and improved performance in certain tests of executive function. PBT2 was safe and well-tolerated.

#### 5. Diverting CSF in Alzheimer's patients

The neuropathological hallmarks of Alzheimer's disease include amyloid plaques and neurofibrillary tangles; these are formed from amyloid peptide and tau protein, respectively. If amyloid and tau are more efficiently cleared from the central nervous system (CNS), there is a theoretically lower likelihood of their deposit in the brain, and hence a lower risk of resultant neurodegeneration. Encouraged by the positive results of a pilot study,[24] Silverberg *et al*.[25] examined whether draining CSF through a ventriculoperitoneal shunt would reduce the macromolecular load on the CNS and benefit patients with Alzheimer's disease. Regrettably, this large (*n* = 215), 9-month, double-blind, controlled study found that a low-flow ventriculoperitoneal shunt neither improves dementia ratings nor slows deterioration in patients with probable Alzheimer's disease.

## References

[1] Kirsch I, Deacon BJ, Huedo-Medina TB, Scoboria A, Moore TJ, Johnson BT (2008). Initial severity and antidepressant benefits: A meta-analysis of data submitted to the Food and Drug Administration. PLoS Med.

[2] Sneed JR, Rutherford BR, Rindskopf D, Lane DT, Sackeim HA, Roose SP (2008). Design makes a difference: A meta-analysis of antidepressant response rates in placebo-controlled versus comparator trials in late-life depression. Am J Geriatr Psychiatry.

[3] Kennedy SH, Andersen HF, Lam RW (2006). Efficacy of escitalopram in the treatment of major depressive disorder compared with conventional selective serotonin reuptake inhibitors and venlafaxine XR: A meta-analysis. J Psychiatry Neurosci.

[4] Lam RW, Andersen HF, Wade AG (2008). Escitalopram and duloxetine in the treatment of major depressive disorder: A pooled analysis of two trials. Int Clin Psychopharmacol.

[5] Papakostas GI, Fava M (2007). A meta-analysis of clinical trials comparing milnacipran, a serotonin--norepinephrine reuptake inhibitor, with a selective serotonin reuptake inhibitor for the treatment of major depressive disorder. Eur Neuropsychopharmacol.

[6] Papakostas GI, Thase ME, Fava M, Nelson JC, Shelton RC (2007). Are antidepressant drugs that combine serotonergic and noradrenergic mechanisms of action more effective than the selective serotonin reuptake inhibitors in treating major depressive disorder? A meta-analysis of studies of newer agents. Biol Psychiatry.

[7] McGeer PL, Schulzer M, McGeer EG (1996). Arthritis and anti-inflammatory agents as possible protective factors for Alzheimer's disease: A review of 17 epidemiologic studies. Neurology.

[8] Szekely CA, Thorne JE, Zandi PP, Ek M, Messias E, Breitner JC (2004). Nonsteroidal anti-inflammatory drugs for the prevention of Alzheimer's disease: A systematic review. Neuroepidemiology.

[9] ADAPT Research Group (2008). Cognitive function over time in the Alzheimer's Disease Anti-inflammatory Prevention Trial (ADAPT): Results of a randomized, controlled trial of naproxen and celecoxib. Arch Neurol.

[10] ADAPT Research Group (2007). Naproxen and celecoxib do not prevent AD in early results from a randomized controlled trial. Neurology.

[11] Andrade C, Singh NM, Thyagarajan S, Nagaraja N, Rao NS, Chandra JS (2008). Possible glutamatergic and lipid signalling mechanisms in ECT-induced retrograde amnesia: Experimental evidence for involvement of COX-2 and review of literature. J Psychiatr Res.

[12] Andrade C, Thyagarajan S, Singh NM, Vinod PS, Rao NS, Chandra JS (2008). Celecoxib as an in vivo probe of the mechanisms underlying retrograde amnesia in an animal model of ECT. J Neural Transm.

[13] Wolozin B, Wang SW, Li NC, Lee A, Lee TA, Kazis LE (2007). Simvastatin is associated with a reduced incidence of dementia and Parkinson's disease. BMC Med.

[14] Sparks DL, Kryscio RJ, Sabbagh MN, Connor DJ, Sparks LM, Liebsack C (2008). Reduced risk of incident AD with elective statin use in a clinical trial cohort. Curr Alzheimer Res.

[15] Cramer C, Haan MN, Galea S, Langa KM, Kalbfleisch JD (2008). Use of statins and incidence of dementia and cognitive impairment without dementia in a cohort study. Neurology.

[16] Lichtenthaler SF, Haass C (2004). Amyloid at the cutting edge: Activation of alpha-secretase prevents amyloidogenesis in an Alzheimer disease mouse model. J Clin Invest.

[17] Morris MC, Evans DA, Bienias JL, Tangney CC, Bennett DA, Wilson RS (2003). Consumption of fish and omega-3 fatty acids and risk of incident Alzheimer disease. Arch Neurol.

[18] Huang TL, Zandi PP, Tucker KL, Fitzpatrick AL, Kuller LH, Fried LP (2005). Benefits of fatty fish on dementia risk are stronger for those without APOE epsilon. Neurology.

[19] Nurk E, Drevon CA, Refsum H, Solvoll K, Vollset SE, Nygerd O (2007). Cognitive performance among the elderly and dietary fish intake: The Hordaland Health Study. Am J Clin Nutr.

[20] van Gelder BM, Tijhuis M, Kalmijn S, Kromhout D (2007). Fish consumption, n-3 fatty acids, and subsequent 5-y cognitive decline in elderly men: The Zutphen Elderly Study. Am J Clin Nutr.

[21] van de Rest O, Geleijnse JM, Kok FJ, van Staveren WA, Dullemeijer C, OldeRikkert MG (2008). Effect of fish oil on cognitive performance in older subjects: A randomized, controlled trial. Neurology.

[22] Wisniewski T, Konietzko U (2008). Amyloid-beta immunisation for Alzheimer's disease. Lancet Neurol.

[23] Lannfelt L, Blennow K, Zetterberg H, Batsman S, Ames D, Harrison J (2008). Safety, efficacy, and biomarker findings of PBT2 in targeting A-beta as a modifying therapy for Alzheimer's disease: A phase IIa, double-blind, randomised, placebo-controlled trial. Lancet Neurol.

[24] Silverberg GD, Levinthal E, Sullivan EV, Bloch DA, Chang SD, Leverenz J et al (2002). Assessment of low-flow CSF drainage as a treatment for AD: Results of a randomized pilot study. Neurology.

[25] Silverberg GD, Mayo M, Saul T, Fellmann J, Carvalho J, McGuire D (2008). Continuous CSF drainage in AD: Results of a double-blind, randomized, placebo-controlled study. Neurology.

